# The dynamic interplay between loneliness and health behaviors from adolescence to midlife: A Norwegian population-based cohort study

**DOI:** 10.1017/S0033291726105029

**Published:** 2026-08-03

**Authors:** Libor Potočár, Laura Cachón Alonso, Michal Kozák, Rubén Rodríguez-Cano, Tomáš Formánek, Tilmann von Soest

**Affiliations:** 1 PROMENTA Research Center, Department of Psychology, University of Oslo, Oslo, Norway; 2 Department of Psychology and Logopedics, Faculty of Medicine, University of Helsinki, Helsinki, Finland; 3 Norwegian Social Research (NOVA), Oslo Metropolitan University, Oslo, Norway; 4 Department of Psychology, Norwegian University of Science and Technology (NTNU), Trondheim, Norway; 5 Department of Clinical Epidemiology, Aarhus University and Aarhus University Hospital, Aarhus, Denmark; 6 National Centre for Register-based Research, Department of Public Health, Aarhus University, Aarhus, Denmark

**Keywords:** loneliness, health behaviors, smoking, alcohol use, physical exercise, body mass index, adolescence, midlife

## Abstract

**Background:**

Adverse health behaviors, such as physical inactivity and smoking, constitute a potential pathway connecting loneliness to poor mental and physical health. No prior study has investigated the dynamic interplay between loneliness and a broad range of health behaviors across the first half of life.

**Methods:**

We used data from a Norwegian population-based cohort study (*n* = 3,072) spanning 28 years from adolescence to midlife. Applying a propensity score-based causal inference approach, we examined bidirectional prospective associations between loneliness and health behaviors, comprising alcohol use, body mass index (BMI), smoking, and physical exercise.

**Results:**

Loneliness in adolescence was associated with lowered physical exercise (mean difference: −14.13 minutes/week, 95% CI: −26.54 to −1.72), and loneliness during young adulthood was linked to increased BMI (0.37 points, 0.12–0.63). In the reverse direction, moderate alcohol use (−0.07 SD, −0.13 to −0.01 and − 0.19 SD, −0.33 to −0.05), smoking (−0.12 SD, −0.23 to −0.01), and moderate physical exercise (−0.16 SD, −0.26 to −0.07 and − 0.07 SD, −0.14 to −0.01) during adolescence and emerging adulthood were linked to reduced loneliness. All other associations were consistent with a null effect.

**Conclusions:**

Our results provide limited support for the causal effects of loneliness on health behaviors across the first half of life. By contrast, findings in the reverse direction suggest that health behaviors in young people – whether health-promoting or risky – are socially embedded and may facilitate socializing. To tackle youth loneliness, public health policies should promote equitable access to health-promoting behaviors.

## Introduction

Loneliness has gained increasing recognition as a major societal and public health challenge in recent years (Goldman et al., 2024). Previous research has demonstrated associations between loneliness and a range of adverse health outcomes, most notably depression (Abdellaoui et al., 2019; Goosby, Bellatorre, Walsemann, & Cheadle, 2013), severe infections (Elovainio et al., 2023), cardiovascular disease (Abdellaoui et al., 2019; Hakulinen et al., 2018), and all-cause mortality (Elovainio et al., 2017; Wang et al., 2023; Yu et al., 2023). However, the mechanisms underlying these associations remain incompletely understood.

Adverse health behaviors represent a potential mechanism through which loneliness might affect health (Christiansen, Larsen, & Lasgaard, 2016). The evolutionary theory of loneliness offers a useful theoretical framework, proposing that individuals who feel lonely may display hypervigilance to social threats (Hawkley & Cacioppo, 2010; Qualter et al., 2015). Over time, this sustained alertness may impair executive functioning and self-regulation, thereby heightening susceptibility to impulsive coping strategies and maladaptive health behaviors (Hawkley & Cacioppo, 2010). Consistent with this perspective, cohort studies in older adults and genetic evidence demonstrate that loneliness is associated with physical inactivity (Hawkley, Thisted, & Cacioppo, 2009; Pengpid, Peltzer, & Anantanasuwong, 2023; Shankar, McMunn, Banks, & Steptoe, 2011), smoking (Shankar et al., 2011; Wootton et al., 2021), and underweight (Pengpid et al., 2023). Among adults experiencing loneliness, such adverse health behaviors might contribute to multiple negative health outcomes, including an increased risk of depression (Liang et al., 2024), severe infections (Elovainio et al., 2023), cardiovascular disease (Hakulinen et al., 2018; Liang et al., 2024), obesity (Liang et al., 2024), and premature mortality (Elovainio et al., 2017). However, recent cohort studies of older adults that adjusted for a broad range of confounders have found no evidence for links between loneliness and subsequent physical activity (Hong et al., 2023; Kobayashi & Steptoe, 2018; Schrempft, Jackowska, Hamer, & Steptoe, 2019), alcohol use (Hong et al., 2023; Kobayashi & Steptoe, 2018), smoking (Hong et al., 2023; Kobayashi & Steptoe, 2018), and overweight (Hong et al., 2023; Kobayashi & Steptoe, 2018). These findings suggest earlier findings may partly reflect bias, such as residual confounding. Moreover, research on loneliness-health behavior linkages predominantly focused on older adults (Hawkley & Capitanio, 2015; Kobayashi & Steptoe, 2018), failing to systematically examine these behavioral precursors of disease and disability across the first half of life.

Adolescence is a crucial developmental period when future adult behaviors are dynamically shaped, and in which loneliness is particularly prevalent (Luhmann & Hawkley, 2016; von Soest, Luhmann, & Gerstorf, 2020). However, findings from older adult populations may not directly generalize to earlier life stages, as risky health behaviors such as alcohol use, physical exercise, and smoking in adolescence and emerging adulthood are often socially embedded and shaped by peer norms, social network processes, and identity formation (Montgomery et al., 2020), potentially reducing the relative importance of loneliness as an independent driver of these behaviors. Across early life stages, prior research has linked loneliness and lack of social connections to poorer self-rated health and cardiometabolic risk factors such as high blood pressure, elevated body mass index (BMI), cholesterol, and inflammation (Goosby et al., 2013; Rangul et al., 2025; Yang et al., 2016). A large-scale Danish study further demonstrated associations between loneliness and health behaviors among young people (Jensen et al., 2025); yet its cross-sectional design precluded it from establishing the temporal ordering and directionality of these associations. By contrast, a recent US longitudinal cohort study applying a causal inference framework found no support for causal associations between adolescent loneliness and multiple health behaviors in adulthood, including smoking, alcohol use, physical activity, and overweight/obesity (Kim, Wilkinson, Holt-Lunstad, & VanderWeele, 2025). Such causal inference approaches involve adjustment for preexisting levels of the exposure, the outcome, and a wide range of confounders to minimize the risk of reverse causality and residual confounding (Mulder, Usami, & Hamaker, 2025; VanderWeele, 2017, 2021), consequently providing more robust insights into potential causal linkages. However, systematic investigations into links between loneliness and adverse health behaviors across the first half of life – from adolescence through emerging adulthood and young adulthood – remain scarce.

The reverse direction has received even less attention, despite prior work suggesting that adverse health behaviors may shape social connections and loneliness. For example, as certain health behaviors such as smoking have become increasingly more stigmatized, individuals who engage in these behaviors face social exclusion and marginalization (Christakis & Fowler, 2008; von Soest & Pedersen, 2014). Supporting this notion, a British cohort study demonstrated that smoking was prospectively associated with increased loneliness in older adults (Philip et al., 2022). Mendelian randomization studies further showed bidirectional loneliness–smoking effects (Wootton et al., 2021), as well as the effects of body fat and BMI on loneliness (Abdellaoui et al., 2019). In contrast, some evidence suggests that some health behaviors may also have protective effects on psychosocial outcomes. A US cohort study employing causal inference methodology showed that moderate alcohol use in young adulthood was associated with a lower risk of depression in midlife (Visontay et al., 2023), while an Australian household panel study showed reciprocal linkages between physical activity and loneliness (Groß & Haffa, 2025). Together, these findings suggest that health behaviors may influence psychosocial functioning in complex and potentially bidirectional ways across the life course. Yet, no study has systematically investigated the dynamic interplay between loneliness and a broader range of health behaviors across the first half of life.

To address these evidence gaps, we applied a causal inference framework to data from a Norwegian population-based cohort spanning 28 years from adolescence to midlife, investigating prospective reciprocal associations between loneliness and key health behaviors, comprising alcohol use, BMI, smoking, and physical exercise. We hypothesized that loneliness would be prospectively associated with adverse health behaviors, and that these behaviors, in turn, would be linked to subsequent levels of loneliness.

## Methods

### Open science practices

The study was preregistered on the Open Science Framework (OSF) before conducting statistical analyses (OSF, 2025). To inform the study design, we inspected frequencies and distributions of loneliness and health behavior measures before preregistration. All deviations from the preregistered protocol are documented in the Supplementary Materials (p. 3).

### Procedure and participants

This study is based on the population-based cohort study Young in Norway. Data were collected at five time points across 28 years: 1992 (T0, middle adolescence, mean age 15 years), 1994 (T1, late adolescence, 17 years), 1999 (T2, emerging adulthood, 22 years), 2005 (T3, young adulthood, 28 years), and 2020 (T4, middle adulthood, 43 years). At T0, a stratified, two-stage cluster probability sampling method was employed to draw a nationally representative sample of Norwegian adolescents aged mainly 13–18 years from 67 junior and senior high schools (*n* = 4,187; initial response rate [RR] = 97.0%). Participants were followed up at T1 (*n* = 3,844; RR = 91.8%), T2 (*n* = 2,923; RR = 83.8%), and T3 (*n* = 2,890; RR = 82.4%). Finally, at T4, 2,215 individuals participated (RR = 85.6%, cumulative RR = 52.9%). The present analytical sample encompassed 3,072 individuals responding to at least three data collection waves. A detailed participant flow chart is provided in the Supplementary Materials (Supplementary Figure S1).

To account for potential attrition bias and preserve representativeness of the sample, we performed all analyses using nonresponse weights using inverse probability weighting with a LASSO-selected set of baseline predictors (for details, see Supplementary Materials pp. 4–5). Missing data were handled with multiple imputations by chained equations using the R package *mice* (version 3.16.0). We generated 10 imputed datasets and pooled the estimates using Rubin’s rules.

All procedures adhered to the Helsinki Declaration, and we obtained informed consent from participants throughout the study. The Young in Norway Study was approved by the Norwegian Data Inspectorate and the Regional Committees for Medical and Health Research Ethics (reference 25462).

### Measures

#### Loneliness

At all five waves, loneliness was assessed by a four-item short version of the revised UCLA Loneliness scale (Russell, Peplau, & Cutrona, 1980) (example item: ‘*People are around me but not with me*’). Responses were recorded on a four-point rating scale, ranging from ‘*Never*’ to ‘*Often*’. Composite mean scores were calculated and standardized (mean = 0, standard deviation [SD] = 1) to facilitate interpretation. McDonald’s omega (*ω*) ranged from 0.66 at T1 to 0.81 at T4, indicating satisfactory to very good scale reliability.

#### Health behaviors

Self-reported information on health behaviors was collected at each time point. Alcohol use was measured by multiplying drinking frequency over the past 4 weeks (‘*How many times during the last 4 weeks have you been drinking more than a few sips of alcohol?*’) by the number of drinks consumed on the last drinking occasion (‘*Last time you drank alcohol, how many drinks [standard units] did you have?*’), yielding an index of average 4-week alcohol use. Smoking behavior was assessed by one multiple-choice item (‘*Do you smoke?*’) and categorized as current smoking (daily [‘*I smoke daily, about … cigarettes*’] or occasional [‘*I smoke, but not daily*’]) versus nonsmoking (all other response options). BMI was calculated based on self-reported weight and height as weight (kg)/height (*m*)^2^. Physical exercise was measured from T2 to T4 by the item ‘*How many hours did you spend on physical exercise last 7 days? (hours/minutes)*’. At T0 and T1, participants reported 7-day frequency of three types of physical activity: ‘*training with a sports team*’, ‘*exercise at a fitness center*’, and individual physical activity (e.g. ‘*jogging*’ and ‘*swimming*’), and we calculated a sum of these activities. To increase consistency, physical exercise measures at T0 and T1 were transformed by multiplying the number of physical activities by 60 (minutes), rendering a harmonized indicator of physical exercise.

#### Confounders

To increase the confidence that the exchangeability assumption holds (i.e. no unmeasured confounding), we selected a comprehensive set of baseline and time-varying confounders based on previous studies on loneliness and health (Kim et al., 2025; Kobayashi & Steptoe, 2018; Pengpid et al., 2023; Shankar et al., 2011) and theoretical considerations. Baseline, time-invariant confounders (*C*_0_, measured at T0) included sociodemographic characteristics (age, gender, migration background, and parental education), early family environment (parental warmth, overcontrol, and behavioral monitoring), parental health behaviors (parental alcohol use and smoking), and chronic health conditions (physical disability, asthma, and reading/writing disability). Time-varying confounders (*L_t_*_-1,_ measured from T0 to T3) comprised socioeconomic characteristics (employment status and educational attainment), indicators of social integration (living situation, relationship status, and number of friends), perceived social support, and depressive symptoms. For a directed acyclic graph showing the assumed causal structure, see Supplementary Figure S2, and detailed information on confounder measurement is provided in the Supplementary Materials (p. 6).

#### Statistical analysis

To examine reciprocal longitudinal associations between loneliness and health behaviors from adolescence to midlife, we applied structural mean modeling (SMM) (Mulder et al., 2025). This causal modeling approach, developed within the potential outcomes framework, is a propensity-score-based, doubly robust modeling approach that allows estimation of causal effects. By accounting for previous levels of loneliness and health behaviors, as well as a comprehensive set of baseline and time-varying confounders, SMM allowed us to minimize the possibility of reverse causation and residual confounding, and examine the effect of *changes* in exposure on subsequent *changes* in the outcome. Because links between health behaviors and psychosocial outcomes may be nonlinear, with adverse outcomes potentially emerging at both very high and very low levels of behavior, a U- or J-shaped relationship is plausible (Visontay et al., 2023). Continuous health behaviors (alcohol use, BMI, and physical exercise) were therefore modeled using natural (restricted cubic) splines, a semi-parametric approach widely used in biomedical research for capturing complex, nonlinear dose–response relationships (Desquilbet & Mariotti, 2010; Ho & Cole, 2023). A detailed description of the overall modeling strategy is provided in the Supplementary Materials (p. 7).

We note that while our analytical approach does not completely rule out the possibility of residual confounding, it greatly strengthens our confidence in causal inference. We considered a comprehensive set of confounders, which also serve as proxies for unmeasured confounders (e.g. indicators of early family environment and parenting as proxies for unobserved confounders such as genetic vulnerability), making the fulfillment of the exchangeability assumption more plausible. For a thorough discussion of the identifiability assumptions required for causal interpretation, including exchangeability, consistency, and correct model specification, see pp. 8–9 in the Supplementary Materials.

We examined prespecified exposure contrasts informed by the WHO (World Health Organization, 2020, 2024) and US (National Institute on Alcohol Abuse and Alcoholism, 2024) guidelines:

1. smoking (daily or occasional) versus nonsmoking (dichotomized);
2. adiposity (BMI = 30) and underweight (BMI = 17) versus normative BMI (BMI = 21.75) (continuous);
3. abstinence (0 units/4 weeks) and heavy alcohol use (60 units/4 weeks) versus moderate drinking (10 units/4 weeks) (continuous);
4. moderate physical exercise (150 minutes/week) versus no physical exercise (0 minutes/week) (continuous); and
5. high loneliness level (1 SD above the mean) versus the mean level (SD = 0) (continuous).

For each contrast, we calculated average effect estimates and 95% confidence intervals (CIs) based on robust (sandwich) standard errors. We additionally tested for moderation by gender and report gender-specific effect estimates.

All data preprocessing and statistical analyses were performed using the software R (version 4.2.3). We used the *splines* package (version 4.2.3) to model nonlinear dose–response relationships and the *marginaleffects* package (version 0.24.0) for computing effect estimates.

#### Sensitivity analyses

Several sensitivity analyses assessed the robustness of our findings. First, we reestimated models excluding social isolation, social support, and depressive symptoms from the set of confounders. Second, we assessed associations using heavy episodic drinking rather than total alcohol use. Third, we applied life stage-specific exposure contrasts when examining the effects of health behaviors. Specifically, given that the same level of health behaviors may reflect different substantive meanings across life stages (e.g. the number of alcoholic drinks in adolescence versus in young adulthood), we additionally specified SD-based exposure contrasts separately for each life stage (see Supplementary Table S4). Fourth, we replicated analyses using a direct single-item loneliness measure (‘*I feel lonely*’). Full rationale and technical details of the sensitivity analyses are provided in the Supplementary Materials (p. 10).

## Results

At baseline (T0), the sample comprised 45.3% men and 54.7% women, with a mean age of 15.0 years (SD = 1.9). Most respondents had parents born in Norway (97.9%), while 2.1% had a migration background. Regarding parental educational attainment, 42.6% had parents with a higher education degree. A total of 3.0% had both parents currently unemployed. On average, participants engaged in 167.3 (SD = 157.0) minutes of physical exercise during the past week; consumed 7.4 (SD = 30.5) standard alcohol units in the past 4 weeks, and 19.4% were current smokers. The mean BMI was 20.0 (SD = 2.7), and the mean unstandardized loneliness score was 1.8 (SD = 0.5). Detailed sample characteristics at T0 are presented in Table 1. Descriptive statistics for loneliness and health behaviors from T1 to T4 are shown in Supplementary Tables S1 and S2 in the Supplementary Materials, and missing data statistics are displayed in Supplementary Table S3.

**Table 1.** Sample characteristics at the baseline (T0, adolescence) Table 1. long description.

Gender, *n* (%)	Women	1679 (54.65)
	Men	1393 (45.35)
Age, mean (SD)		14.95 (1.88)
Migration background, *n* (%)	Norway-born parents	2957 (97.88)
	Migrant	64 (2.12)
Chronic health condition, *n* (%)		424 (13.80)
Parental education, *n* (%)	College/university	1262 (42.62)
	Lower level	1699 (57.38)
Parental employment, *n* (%)	Employed	2924 (96.98)
	In unemployment	91 (3.02)
Behavioral monitoring, mean (SD)		2.14 (0.89)
Psychological overcontrol, mean (SD)		2.10 (0.56)
Cold parenting, mean (SD)		1.83 (0.53)
Parental alcohol use, mean (SD)	Abstinence	337 (11.30)
	Heavy use	393 (13.18)
	Low use	2252 (75.52)
Parental smoking, *n* (%)	Daily smoking	1319 (48.89)
	Nonsmoking	1379 (51.11)
Depressive symptoms, mean (SD)		1.71 (0.56)
Social support, mean (SD)		0.23 (0.50)
Living situation, *n* (%)	Not with both parents	695 (23.04)
	With both parents	2322 (76.96)
Relationship status, *n* (%)	In a relationship	602 (19.66)
	Single	2460 (80.34)
Number of friends, *n* (%)	A lot	2638 (87.93)
Alcohol use, mean (SD)		7.38 (30.53)
Smoking status, *n* (%)	Daily/occasional	572 (19.38)
Physical exercise, mean (SD)		2.79 (2.62)
Body mass index, mean (SD)		20.00 (2.66)
Loneliness, mean (SD)		1.84 (0.54)

*Note:* Statistics are based on the incomplete dataset before multiple imputations.

**Figure 1.**
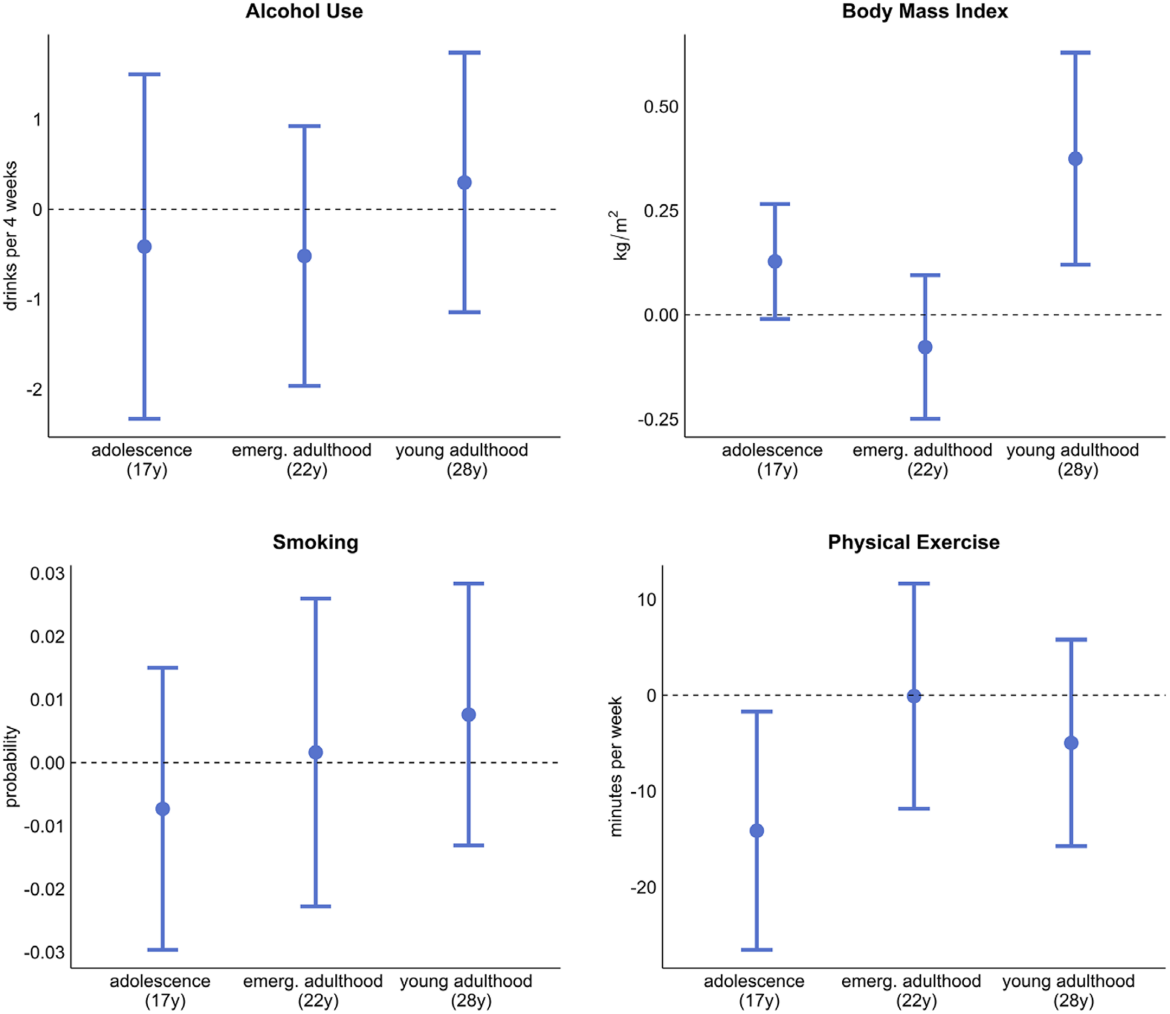
Estimates for causal effects of loneliness on health behaviors. *Note:* Labels of life stages below the panels refer to the exposure period. Figure 1. long description.

In a first step, we examined associations between loneliness and subsequent health behaviors across three life stages: late adolescence, emerging adulthood, and young adulthood. As displayed in Figure 1, a 1 SD increase in loneliness during late adolescence was prospectively associated with a 14-minute reduction in physical exercise (mean difference: −14.13 minutes/week, 95% CI: −26.54 to −1.72). Loneliness during young adulthood was associated with subsequently increased BMI (0.37 points, 95% CI: 0.12–0.63). Loneliness showed no significant effects on smoking and alcohol use, nor on physical exercise and BMI at all other life stages.

**Figure 2.**
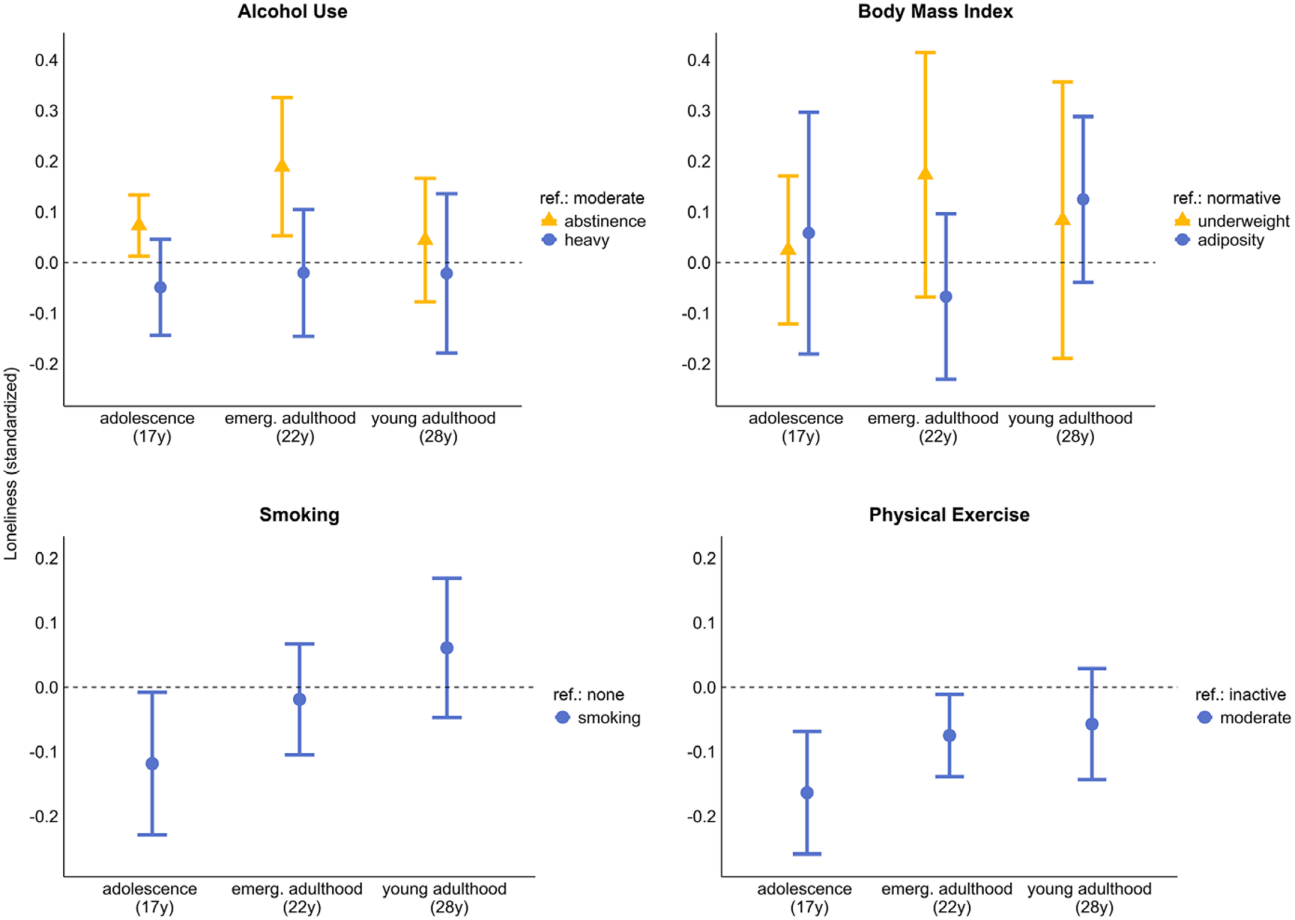
Estimates for causal effects of health behaviors on loneliness. *Note:* Labels of life stages below the panels refer to the exposure period. Figure 2. long description.

Next, we investigated the longitudinal associations between health behaviors and loneliness. As shown in Figure 2, alcohol abstinence during late adolescence and emerging adulthood, compared to moderate alcohol use, was linked to higher levels of subsequent loneliness by 0.07 (95% CI: 0.01–0.13) and 0.19 (95% CI: 0.05–0.33) SDs, respectively. Smoking in late adolescence was prospectively associated with diminished loneliness (−0.12 SD, 95% CI: −0.23 to −0.01). Similarly, moderate physical exercise during adolescence and emerging adulthood was linked to subsequently reduced loneliness levels (−0.16 SD, 95% CI: −0.26 to −0.07; and −0.07 SD, 95% CI: −0.14 to −0.01, respectively). Health behaviors in young adulthood were not associated with later loneliness.

Moderation analyses by gender revealed distinct patterns. Among women, a 1 SD increase in loneliness during late adolescence was linked to a subsequently higher BMI (0.25 points, 95% CI: 0.07 to 0.43), and both underweight in emerging adulthood and adiposity in young adulthood were longitudinally linked to higher subsequent loneliness by 0.27 SD (95% CI: 0.02–0.51) and 0.24 SD (95% CI: 0.06–0.42), respectively. Among men, a 1 SD increase in loneliness during late adolescence was linked to lower levels of physical exercise (−26.29 minutes/week, 95% CI: −43.24 to −9.34), whereas moderate physical exercise in late adolescence was associated with reduced loneliness levels (−0.21 SD, 95% CI: −0.35 to −0.07). Detailed results are shown in Supplementary Figures S4 and S5 in the Supplementary Materials.

Results of sensitivity analyses were largely consistent with the main findings (Supplementary Figures S6–S11 in the Supplementary Materials). Reestimation of models using a reduced set of time-varying confounders revealed only marginal changes in the effect estimates. Notably, exclusion of theoretically relevant confounders, such as prior depressive symptoms, had only a negligible influence on the results, suggesting that our adjustment set was reasonably comprehensive. This pattern further suggests that the predominant null findings were not driven by specific modeling decisions or overadjustment and supports the robustness of the conclusions across alternative model specifications.

Given the substantial number of null findings for associations between loneliness and subsequent health behaviors, we performed post hoc positive control analyses to assess the validity of our analytical approach. Using the same modeling framework, we examined the well-established longitudinal associations between loneliness and depressive symptoms (Goosby et al., 2013; Kim et al., 2025). Results showed a consistent pattern of prospective associations between loneliness and depressive symptoms across all life stages (see Supplementary Figure S12 in the Supplementary Materials), supporting the robustness and sensitivity of our analytical approach to detect effects when present. Bivariate associations (i.e. unadjusted/crude estimates) between loneliness and subsequent health behaviors are shown in Supplementary Figure S13.

## Discussion

In this Norwegian population-based cohort study spanning 28 years, we applied a causal inference approach to investigate the dynamic interplay between loneliness and health behaviors from adolescence to midlife. We showed that increased levels of loneliness in adolescence were longitudinally linked to lower physical exercise, and elevated loneliness in young adulthood was associated with subsequently increased BMI. All other associations between loneliness and subsequent health behaviors were consistent with null effects. In the reverse direction, moderate alcohol use, smoking, and moderate physical exercise in adolescence and emerging adulthood were prospectively linked to lower levels of loneliness. We also observed tentative gender-specific patterns: reciprocal loneliness–BMI associations in women, and bidirectional loneliness-physical exercise linkages among men.

Our findings, providing limited support for causal effects of loneliness on subsequent health behaviors, are broadly in line with a recent US cohort study employing a causal inference framework, reporting no prospective associations between adolescent loneliness and adult health behaviors, including smoking, alcohol use, physical activity, and overweight/obesity (Kim et al., 2025). Similarly, these findings align with longitudinal studies demonstrating no loneliness-health behavior linkages in older adults (Hong et al., 2023; Kobayashi & Steptoe, 2018; Schrempft et al., 2019) and evidence showing weak genetic loneliness-obesity linkages (Liang et al., 2024). However, our findings contrast with longitudinal studies showing that loneliness is longitudinally associated with physical inactivity (Hawkley et al., 2009; Pengpid et al., 2023; Shankar et al., 2011) and smoking (Shankar et al., 2011) among older adults. Two complementary explanations may account for these discrepancies. First, unlike most previous cohort studies, we applied a causal inference approach that adjusted for prior levels of loneliness and health behaviors, as well as a comprehensive set of baseline and time-varying confounders. This approach enabled estimation of the effects of incident increases in loneliness on subsequent changes in health behaviors, thereby reducing the risk of residual confounding and reverse causation, leading to less biased estimates than in conventional approaches. Second, whereas previous studies largely focused on older adults (Hong et al., 2023; Kobayashi & Steptoe, 2018; Pengpid et al., 2023; Shankar et al., 2011), our study examined the first half of life, a period when smoking, alcohol use, and physical exercise are more socially embedded. During adolescence and emerging adulthood, health behaviors may be shaped primarily by peer and social contexts (Montgomery et al., 2020) rather than by internal psychological states such as loneliness. By contrast, in older age, when solitary smoking and alcohol use tend to be more commonly endorsed and socially accepted, loneliness may have a more pronounced effect on these adverse health behaviors.

Given the limited support for the causal effects of loneliness on health behaviors, other pathways may underlie the links between loneliness and subsequent physical health and mortality. In our positive control outcome analysis, elevated loneliness was consistently associated with increases in depressive symptoms across all examined life stages, aligning with prior evidence demonstrating robust links between loneliness and subsequent depressive symptomatology (Goosby et al., 2013; Kim et al., 2025). Importantly, depression and other mental health problems are linked to increased risk of a wide range of adverse physical health conditions (Momen et al., 2020), and these conditions are in turn related to higher mortality among individuals with preexisting mental health conditions (Formánek et al., 2022, 2024). One plausible interpretation is therefore that loneliness in young people may primarily contribute to poorer mental health, which, in turn, may increase vulnerability to adverse physical health outcomes and premature mortality later in life.

When examining the effect of health behaviors on loneliness, we demonstrated that moderate physical exercise, moderate alcohol use, and smoking during adolescence and emerging adulthood were related to lower levels of loneliness. These findings suggest that health behaviors in young people – whether health-promoting or risky – may contribute to reducing loneliness. This pattern contrasts with a recent cohort study in England, showing that smoking among older adults was associated with increased loneliness (Philip et al., 2022). However, our results align with a US cohort study employing a causal inference approach, which demonstrated that moderate alcohol use in young adulthood was associated with a reduced risk of depression (Visontay et al., 2023) and an Australian household panel study showing longitudinal links between physical activity and reduced loneliness (Groß & Haffa, 2025). One potential explanation for these conflicting findings relates again to the social embeddedness of health behaviors. During adolescence and emerging adulthood, behaviors such as smoking, alcohol use, and physical exercise often occur in peer contexts, where they may facilitate socializing, provide social validation, and strengthen a sense of belonging (Montgomery et al., 2020), thereby reducing loneliness. These mechanisms may be less relevant at later life stages. Period and cohort effects may also help to explain the results. Our participants were adolescents in the 1990s, when smoking and alcohol use were common and socially accepted in Norway. In this socio-historical context, engaging in these behaviors may have aligned with prevailing norms and fostered social connectedness. By contrast, as some of these behaviors have become less common and increasingly stigmatized, they may now contribute to social marginalization (Christakis & Fowler, 2008; von Soest & Pedersen, 2014) and heightened loneliness (Philip et al., 2022).

Gender-stratified analyses showed that elevated loneliness during adolescence was related to increased BMI, whereas both underweight in emerging adulthood and adiposity in young adulthood were longitudinally associated with increased loneliness among women. These findings are consistent with longitudinal evidence suggesting an effect of loneliness on BMI (Goosby et al., 2013) and Mendelian randomization evidence indicating effects of BMI on loneliness (Abdellaoui et al., 2019). Given pervasive norms around the female body, women with nonnormative BMI may be more likely than men to experience body dissatisfaction and feelings of shame and guilt, potentially leading to social withdrawal or rejection by others. Such mechanisms may be particularly salient during emerging and young adulthood, when key social and romantic relationships are established. Among adolescent men, consistent with prior evidence (Groß & Haffa, 2025), we showed that loneliness was prospectively associated with decreased levels of physical exercise, whereas moderate physical exercise was linked to lowered loneliness. This suggests that sports and physical activity may serve as important and socially valued arenas for male adolescent socialization.

The present study has several strengths, including the use of nationally representative cohort data spanning nearly three decades from adolescence to midlife, as well as the application of a causal inference approach designed to minimize residual confounding and reverse causation. However, several limitations should be acknowledged. First, the relatively long time intervals between data collection waves might have been suboptimal for capturing short-term causal dynamics (Groß & Haffa, 2025). Longitudinal designs with more frequent assessments may better reflect the temporal fluctuations in loneliness and health behaviors. Second, future research should consider the social context in which health behaviors occur – specifically, whether they are socially or solitarily enacted – as this may moderate their associations with loneliness. Third, while our study examined situational changes in loneliness, more persistent feelings of social disconnection may have stronger and more enduring effects on health. Investigating the long-term impact of chronic loneliness across the first half of life is therefore warranted. Fourth, alcohol use was not legal for most respondents at the first examined life stage (in adolescence, mean age = 17 years). Although we cannot rule out underreporting bias, the pattern of associations with subsequent loneliness at this life stage was largely similar to that in emerging adulthood (mean age = 22 years), when alcohol consumption is legal. This consistency suggests that reporting bias is unlikely to fully explain the observed findings. Fifth, this study was conducted in Norway, an egalitarian welfare state with a high standard of living, which may limit the generalizability to non-Western or less affluent countries. Finally, the single-cohort design precluded disentangling age (e.g. adolescence), period (e.g. the 1990s), and cohort (e.g. Millennials) effects. Future research should therefore examine whether associations vary across birth cohorts, geographical and historical contexts, and examine how shifting social norms influence the relationship between loneliness and health behaviors.

## Conclusion

In this Norwegian population-based cohort study spanning 28 years from adolescence to midlife, we applied a causal inference approach to investigate the dynamic interplay between loneliness and health behaviors. We found little evidence supporting causal pathways from loneliness to subsequent adverse health behaviors across the first half of life. In the opposite direction, moderate alcohol use, smoking, and moderate physical exercise in adolescence and emerging adulthood were prospectively linked to reduced loneliness levels. These findings suggest that health behaviors – whether health-promoting or risky – are socially embedded and may serve as important avenues for socializing in young people, underscoring the importance of a life course approach to interventions targeting social well-being. To address youth loneliness, public health strategies should promote equitable access to health-promoting behaviors that foster social inclusion and belonging. This may include, for example, institutional support for organized sports activities, ensuring that young people have access to inclusive sports environments.

## Supporting information

10.1017/S0033291726105029.sm001 Potočár et al. supplementary material Potočár et al. supplementary material

## Data Availability

Due to the sensitive nature of the data and data protection requirements, the data are not publicly available. The complete analytical R script is available here: https://github.com/libpot/Loneliness_Health_Factors.

## Supplementary material

The supplementary material for this article can be found at http://doi.org/10.1017/S0033291726105029.

## Long descriptions

### Table 1. Long description

The table presents data for a sample of adolescents across several categories.

Demographics and Background:

- Gender: Women 1679 (54.65 percent), Men 1393 (45.35 percent).

- Age: mean 14.95, S D 1.88.

- Migration background: Norway-born parents 2957 (97.88 percent), Migrant 64 (2.12 percent).

- Chronic health condition: 424 (13.80 percent).

- Parental education: College or university 1262 (42.62 percent), Lower level 1699 (57.38 percent).

- Parental employment: Employed 2924 (96.98 percent), In unemployment 91 (3.02 percent).

Parenting and Home Environment:

- Behavioral monitoring: mean 2.14, S D 0.89.

- Psychological overcontrol: mean 2.10, S D 0.56.

- Cold parenting: mean 1.83, S D 0.53.

- Parental alcohol use: Abstinence 337 (11.30 percent), Heavy use 393 (13.18 percent), Low use 2252 (75.52 percent).

- Parental smoking: Daily smoking 1319 (48.89 percent), Nonsmoking 1379 (51.11 percent).

- Living situation: Not with both parents 695 (23.04 percent), With both parents 2322 (76.96 percent).

Psychosocial and Health Behaviors:

- Depressive symptoms: mean 1.71, S D 0.56.

- Social support: mean 0.23, S D 0.50.

- Relationship status: In a relationship 602 (19.66 percent), Single 2460 (80.34 percent).

- Number of friends: A lot 2638 (87.93 percent).

- Alcohol use: mean 7.38, S D 30.53.

- Smoking status: Daily or occasional 572 (19.38 percent).

- Physical exercise: mean 2.79, S D 2.62.

- Body mass index: mean 20.00, S D 2.66.

- Loneliness: mean 1.84, S D 0.54.

Navigate back to Table 1..

### Figure 1. Long description

A multi-panel figure containing four dot plots. Each plot shares a common x-axis with three life stages: adolescence (17y), emerging adulthood (22y), and young adulthood (28y). A horizontal dashed line at zero represents no effect.

1. Top-left panel: Alcohol Use. Y-axis is drinks per 4 weeks ranging from -3 to 1. Adolescence shows a point estimate near -0.5 with wide error bars from -2.3 to 1.5. Emerging adulthood is near -0.5 with bars from -2 to 0.9. Young adulthood is near 0.3 with bars from -1.1 to 1.8.

2. Top-right panel: Body Mass Index. Y-axis is k g / m super 2 ranging from -0.25 to 0.50. Adolescence is near 0.13 with bars from 0 to 0.27. Emerging adulthood is near -0.08 with bars from -0.25 to 0.1. Young adulthood is near 0.38 with bars from 0.12 to 0.63.

3. Bottom-left panel: Smoking. Y-axis is probability ranging from -0.03 to 0.03. Adolescence is near -0.007 with bars from -0.03 to 0.015. Emerging adulthood is near 0.002 with bars from -0.023 to 0.026. Young adulthood is near 0.008 with bars from -0.013 to 0.028.

4. Bottom-right panel: Physical Exercise. Y-axis is minutes per week ranging from -30 to 10. Adolescence is near -14 with bars from -26 to -2. Emerging adulthood is near 0 with bars from -12 to 12. Young adulthood is near -5 with bars from -16 to 6.

Navigate back to Figure 1..

### Figure 2. Long description

A multi-panel figure with four plots arranged in a two-by-two grid. The y-axis for all panels represents Loneliness standardized, ranging from negative 0.2 to 0.4. The x-axis for all panels lists three life stages: adolescence 17y, emerging adulthood 22y, and young adulthood 28y. A horizontal dashed line at 0.0 indicates the null effect.

Top-left panel: Alcohol Use. Reference is moderate. Yellow triangles represent abstinence, showing positive effects on loneliness that peak in emerging adulthood. Blue circles represent heavy use, showing slight negative effects across all stages.

Top-right panel: Body Mass Index. Reference is normative. Yellow triangles represent underweight, showing positive effects with wide error bars. Blue circles represent adiposity, showing a shift from positive in adolescence to negative in emerging adulthood, then positive again in young adulthood.

Bottom-left panel: Smoking. Reference is none. Blue circles represent smoking, showing a linear upward trend from a negative effect in adolescence to a positive effect in young adulthood.

Bottom-right panel: Physical Exercise. Reference is inactive. Blue circles represent moderate exercise, showing negative effects on loneliness across all three life stages, with the strongest negative effect in adolescence.

Navigate back to Figure 2..
