# Supplementary material for: The dynamic interplay between loneliness and health behaviors from adolescence to midlife: A Norwegian population-based cohort study
Source: Psychol Med. 2026 Aug 3;56:e248. doi: 10.1017/S0033291726105029 (PMC13439249; doi:10.1017/S0033291726105029)
Supplement: Supplementary file 1 [file S0033291726105029sup001.docx]

# The dynamic interplay between loneliness and health behaviors from adolescence to midlife: A Norwegian population-based cohort study

Libor Potočár, Laura Cachón Alonso, Michal Kozák, Rubén Rodríguez-Cano, Tomáš Formánek, Tilmann von Soest

# **Supplementary Materials**

**Contents**

[Deviations from the pre-registered research protocol	3](#_Toc230792921)

[Non-response weighting	4](#_Toc230792922)

[Confounder measurement	6](#_Toc230792923)

[Structural mean modeling	7](#_Toc230792924)

[Causal identification assumptions	8](#_Toc230792925)

[Sensitivity analyses	10](#_Toc230792926)

[Tables	11](#_Toc230792927)

[Table S1. Wave-specific descriptive statistics.	11](#_Toc230792928)

[Table S2. Wave-specific scale ranges	12](#_Toc230792929)

[Table S3. Proportions of missing data	13](#_Toc230792930)

[Table S4. Life stage-specific exposure contrasts	14](#_Toc230792931)

[Figures	15](#_Toc230792932)

[Figure S1. Flowchart of the study participation	15](#_Toc230792933)

[Figure S2. Directed acyclic graph	16](#_Toc230792934)

[Figure S3. Specification of the exposure model and outcome model	17](#_Toc230792935)

[Figure S4. Estimates for gender-specific effects of loneliness on health behaviors	18](#_Toc230792936)

[Figure S5. Estimates for gender-specific effects of health behaviors on loneliness	19](#_Toc230792937)

[Figure S6. Estimates for effects of loneliness on health behaviors, unadjusted for time-varying indicators	20](#_Toc230792938)

[Figure S7. Estimates for effects of loneliness on health behaviors, unadjusted for time-varying indicators	21](#_Toc230792939)

[Figure S8. Estimates for effects of binge drinking on loneliness	22](#_Toc230792940)

[Figure S9. Estimates for effects of health behaviors with life-stage specific contrasts	23](#_Toc230792941)

[Figure S10. Estimates for effects of loneliness measured directly on health behaviors	24](#_Toc230792942)

[Figure S11. Estimates for effects of health behaviors on loneliness measured directly	25](#_Toc230792943)

[Figure S12. Estimates for bi-directional effects of loneliness and depressive symptoms	26](#_Toc230792944)

[Figure S13. Bivariate associations between loneliness and health behaviors	27](#_Toc230792945)

[Figure S14. Directed acyclic graph of unmeasured confounding	28](#_Toc230792946)

## Deviations from the pre-registered research protocol

Physical exercise was measured somewhat differently across data collection waves. To allow for direct comparisons of physical exercise measures across waves, activity level at T0 and T1 (measured in terms of sessions per week) was multiplied by 60, representing an estimated average duration of one session (in minutes), rendering a directly comparable index of physical exercise across all waves.

To address potential confounding due to chronic health conditions, we additionally included a baseline covariate (T0) indicative of the presence of physical disability, asthma, or reading/writing disability.

Pre-specified marginal contrast for health behaviors showed low occurrence at some measurement waves (e.g., BMI of 30 at T1; late adolescence). Furthermore, a consistent definition of marginal contrasts would potentially represent inconsistent qualities across life stages. For instance, heavy alcohol use defined as 60 standard drinks would be arguably indicative of more problematic alcohol use in late adolescence than during young adulthood. To better align with the life course perspective, we additionally defined in sensitivity analyses the following life stage-specific exposure contrasts (see Table S4): 1) +1 standard deviation (SD) and abstinence (0 standard units) vs. mean for alcohol use, 2) +1 SD and -1 SD vs. mean for BMI, 3) mean vs. inactivity (0 minutes) for physical exercise.

To facilitate comparison with total alcohol consumption used in the main analyses, we specified exposure contrasts for heavy episodic drinking (i.e., binge drinking) in sensitivity analyses as follows: 1) heavy episodic drinking at least once a month vs. occasional binge drinking (i.e., 2-5 times a year), and 2) no binge drinking vs. occasional binge drinking.

When examining the effect of loneliness on subsequent health behaviors, we computed only the contrasts for 1 SD increase relative to the mean loneliness value. We did so because we used a simple linear functional form for loneliness, and reporting contrasts for 1 SD increase compared to both the mean and 1 SD below the mean would be redundant.

Given the unexpected extent of null findings for the associations between loneliness and subsequent health behaviors, we performed positive control analyses to assess whether the approach is able to detect empirically and theoretically well-established effects. We did so by examining linkages between loneliness and depressive symptoms.

## Non-response weighting

To account for potential selection bias resulting from differential attrition and to maintain the initial sample's representativeness, we used non-response weights based on inverse probability weighting (IPW). We proceeded in the following steps^1,2^: 1) identifying potential baseline predictors of longitudinal study participation, 2) selecting variables via least absolute shrinkage and selection operator (LASSO) regression, and 3) estimating stabilized non-response weights via logistic regression and trimming.

First, in the baseline sample (T0; n = 4,026), we identified 89 measures organized into 8 overarching context themes. For measurement scales, composite mean scores were calculated. Continuous variables were transformed by dividing them into five percentile-based groups (i.e., quintiles). In case of rare occurrences in categorical or continuous variables (e.g., substance use frequency or number of cinema visits in the past week), categories were merged into fewer levels to improve precision. Given that missing values in covariates are often likely associated with the undisclosed true value (e.g., illicit drug use), an extra category of missing values was defined for each covariate.

Second, we constructed a binary variable indicating whether participants showed longitudinal participation (n = 3,072) or dropped out during the course of the study. To select variables with the highest prediction power of the longitudinal study participation, we fitted a multivariable regression LASSO model separately for each context theme. LASSO represents a regularization machine learning method that shrinks unstable predictors to zero by imposing a penalty to regression coefficients, enabling an effective variable selection^3^. The tuning parameter (lambda; λ), which controls the strength of the penalty, was selected using a 10-fold cross-validation.

Third, we computed the probability of longitudinal study participation using logistic regression with a LASSO-identified set of baseline predictors. Then, we calculated the non-response weights, and to control extreme weights' influence, we performed trimming at 1^th^ and 99^th^ percentiles^4^. For LASSO regression and IPWs construction, we used *glmnet* (version 4.1-8) and *WeightIt* (version 1.1.0) R packages, respectively.

Applying LASSO regression across 8 context themes, we selected the following set of baseline predictors of longitudinal participation: gender, dyslexia, religiosity, parental educational attainment, parental occupational status, and parental alcohol use. Construction of non-response weights using the above-mentioned baseline variables substantially improved covariate balance across dropouts and longitudinal participants (r < 0.10 for all). When using non-response weights, the mean magnitude of differences in previously unbalanced covariates dropped from 8.1% to 2.2%, representing a 73% decrease in potential bias.

For further details on methodological procedure and results, see a previous study that constructed non-response weights in the Young in Norway cohort^5^.

## Confounder measurement

The baseline confounders (C_0_, measured at T0) included:

1. Sociodemographic characteristics: age (continuous: in years), gender (binary: women/men), ethnic background (binary: Norway-born/migrant), parental educational attainment (binary: college or university/lower).
2. Early family environment: Parental warmth and psychological overcontrol were assessed using two 5-item subscales from the Parental Bonding Instrument (PBI; continuous: composite mean score)^6,7^, and parental behavioral monitoring was measured by a 6-item instrument (continuous: composite mean score).
3. Parental health behaviors: alcohol use (categorical: abstinence/low-to-moderate use/heavy use), and parental smoking (binary: daily smoking/not).
4. Chronic health condition (binary: yes, if physical disability, asthma, or reading/writing disability present).

Time-varying confounders (L_0-3_, measured from T0 to T3) were conceptualized as follows:

1. Sociodemographic characteristics: employment status (parental at T0 and T1; binary: full-time or part-time employment/unemployed, and individual’s own from T2 to T4; categorical: full-time or part-time employment/unemployed/economically inactive) and educational attainment (from T2 to T4; binary: college or university/lower).
2. Social isolation factors: living situation (binary: not living with parents at Wave 0 and 1 and living alone from T2 to T4 = 1), relationship status (binary: single = 1), and number of friends (binary: a lot = 1).
3. Perceived social support was assessed by asking respondents to list available social support providers in four hypothetical situations (e.g., dealing with a personal problem; continuous: composite mean score)^8^.
4. Depressive symptomatology was measured using a 6-item Depressive Mood Inventory (DMI)^9^ on a 4-point Likert-type scale (continuous: composite mean score).

## Structural mean modeling

We fitted two sets of generalized linear models (GLM) by switching the exposure and outcome pair. First, we sequentially analyzed the prospective associations between loneliness and subsequent health behaviors, and, second, prospective relationships between health behaviors and subsequent loneliness (see Figure S2 for a directed acyclic graph depicting the assumed causal structure). For each exposure (Xt), an exposure model was specified with baseline confounders (C_0_) and preceding time-varying confounders (Lt-_1_), prior outcome (Yt-_1_), and prior exposure (Xt-_1_) (see Panel A in Figure S3). Then, conditional densities or propensity scores for binary exposures were calculated (PSx_t_). These predicted values, along with all the covariates from the exposure model, were then included in the outcome model, and an association of the observed exposure (Xt) with the subsequent outcome (Yt_+1_) was estimated (see Panel B in Figure S3).

To justify the assumption of sequential conditional exchangeability (i.e., no unmeasured confounding at each time point) necessary for causal inference in structural mean models (SMM)^10^, we assume that the included baseline and time-varying confounders are sufficient to ensure the conditional independence of exposure and potential outcomes, and, thus, to identify a causal effect of interest. For a thorough discussion of underlying causal assumptions, see pp. 8-9.

We applied linear regression models for modeling loneliness and BMI, negative binomial models for alcohol use and physical exercise, and logistic models for smoking status. Because adverse health behaviors (e.g., alcohol use) may translate to positive psychosocial outcomes at moderate levels but adverse psychosocial outcomes if exceedingly high (e.g., heavy alcohol use) or very low/zero (abstinence), a U- or J-shaped relationship seems likely^11^. Continuous health behaviors (alcohol use, BMI, and physical exercise) were therefore modeled using natural (restricted cubic) splines, a semi-parametric approach widely used in biomedical research for capturing complex, non-linear dose-response relationships ^12,13^. To allow for sufficient flexibility to capture curvilinearity in the data and avoid over-fitting, natural splines were specified with 3 equally spaced inner knots for alcohol use and BMI, and 1 knot for physical exercise. By imposing linear constraints beyond boundary knots, this technique minimizes the influence of outliers (e.g., excessively high levels of alcohol use). We used a simple linear functional form for modeling loneliness as exposure.

## Causal identification assumptions

Causal interpretations of our estimates depend on the fulfillment of multiple causal identification assumptions, including 1) consistency, 2) exchangeability, 3) correct model specification, and 4) no interference^14–17^. Below, we discuss the plausibility of these assumptions in our study:

1. The consistency assumption implies that the model’s exposure variable is well-defined, and that if there are various exposure versions, they have equivalent effects^16,18^. In this study, measurements of alcohol consumption and physical exercise represent a threat to this assumption. In the case of alcohol consumption, the indicator does not differentiate between different beverages consumed (e.g., spirits, beer, wine) or the setting in which alcohol is consumed (i.e., solitary vs. social). Regarding physical exercise, it was measured at the first two waves (T0 and T1) by summing items tapping into different sports activities with varying levels of social engagement (i.e., training with a sports team, in a sports center, on your own) and from T2 to T4 by the item “*How many hours did you spend on physical exercise last 7 days? (hours/minutes)*”. These various facets of alcohol use and different operationalizations of physical exercise could have had different effects on subsequent loneliness levels, compromising the consistency assumption. Future research would benefit from examining the context in which health behaviors occur–specifically, whether they are social or solitary enacted–as this may influence their associations with subsequent loneliness.

Moreover, loneliness is a subjective self-report measure, which makes its definition challenging. We drew upon a standardized 4-item version of the UCLA scale, which taps into different manifestations of feelings of loneliness (i.e., feeling left out, lacking companionship, having nobody to talk to, having people around but not with oneself). To assess potential biases introduced by using this operationalization of loneliness, sensitivity analysis was conducted using an alternative direct or explicit measure of loneliness (one-item measure: “I feel lonely”), largely replicating the main results. However, we cannot rule out the possibility that the same score of loneliness may capture different facets and reasons for these subjective perceptions (e.g., social withdrawal, social exclusion, low-quality relationships), which might have a differential effect on the health outcomes.

2. The exchangeability assumption requires no unmeasured confounding. In this study we aimed to estimate the causal effect of loneliness on health behaviors, and vice versa, by accounting for a comprehensive set of baseline, time-invariant confounders (C_0_, socio-demographic characteristics, indicators of early family environment, parental health behaviors, chronic health conditions) and time-varying confounders (L_t-1_, depressive symptomatology, perceived social support, indicators of objective social isolation and socioeconomic indicators), selected based on previous literature and theoretical assumptions. This analytical approach strengthens causal inference, minimizing potential residual confounding and risk of reverse causation. Although we did not have information on several potentially relevant confounders (such as genetic vulnerability and personality traits), we accounted for a broad range of factors that often serve as proxies for these unobserved sources of confounding (such as indicators of family environment, or prior levels of loneliness and health behaviors). As demonstrated by VanderWeele (2019), adjusting for measured confounders C that are downstream of unmeasured causes can still reduce confounding between the exposure A and outcome Y, even when the unmeasured confounder U is not directly observed (see Figure S14). For example, while familial confounding may explain the associations between offspring's loneliness and subsequent health behaviors, a comprehensive adjustment for measured confounders (such as indicators of family environment) helps to indirectly address such unobserved confounders. Moreover, because we accounted for prior levels of loneliness and health behaviors, the effects of the unobserved confounders would need to affect the examined loneliness-health behaviors links directly (i.e., through pathways not involving the prior levels of loneliness and health behaviors). Nevertheless, residual confounding arising from omitted or unidentified confounders remains possible and should be considered when interpreting the findings.

3. Correct model specification*.* Notably, SMMs represent doubly robust models; thus, the model estimates are unbiased if either the outcome or the exposure (i.e., treatment assignment process) models are correctly specified^20,21^. Moreover, to account for complex non-linear dose-response relationships between continuous exposures and outcomes, we implemented natural splines, which allow sufficient flexibility in capturing the curvilinearity.

4. No interference or SUTVA (stable unit treatment value assumption), assumes that participants’ potential outcomes depend only on their own exposure, ruling out peer effects. Participants were recruited using a stratified, two-stage, school-cluster probability sampling strategy. Thus, at T0 (1992, mean age = 15 years), they were nested within their initial schools and subsequently moved across various educational, job-, and family-related contexts across the 28-year follow-up period. Thus, while some interference might have occurred at T1 (1994), participants’ initial schools and classmates had likely an increasingly diminishing influence across the years.

## Sensitivity analyses

To examine the robustness of our findings, we performed several sets of sensitivity analyses. First, we analyzed the effects of loneliness on health behaviors by re-running all analyses while excluding the time-varying indicators of social isolation (relationship status, living situation, and number of friends) and perceived social support from the adjustment set. Moreover, in a separate model, we additionally excluded depressive symptoms from the adjustment set as well. We performed these analyses to ensure that the results are not due to overlapping constructs between the confounders and the exposure (i.e., loneliness).

Second, to assess the robustness of associations concerning alcohol use, we additionally considered patterns of alcohol consumption using an item on the frequency of heavy episodic drinking (i.e., binge drinking) in the past 12 months (“*[How many times have you] drunk so much that you clearly felt drunk?*”) rated on 6-point Likert-like scale ranging from “*0 times*” to “*More than 50 times*”. Informed by U.S. reference guidelines^22^, we computed exposure contrasts for heavy episodic drinking at least once/month vs. occasional binge drinking (2-5 times/year), and no heavy episodic drinking vs. occasional binge drinking.

Third, considering that the same value on variables related to health behaviors might represent different underlying qualities across life stages (e.g., 60 alcoholic drinks during late adolescence and young adulthood), and due to the low occurrence of observations at pre-specified levels at some waves (e.g., BMI = 30 in emerging adulthood), we additionally adopted a life stage-specific approach to specifying contrasts for health behaviors, comparing SD-based differences to mean and/or 0 at each life stage (see Table S4 for details of exposure contrast specification).

Fourth, if directly asked about feelings of loneliness, people may underreport these due to stigma and social desirability bias. On the other hand, such direct or explicit measures of loneliness may tap more into chronic loneliness, potentially leading to stronger associations with health behaviors^23^. To test this, we additionally operationalized loneliness using a 1-item measure (“*I feel lonely*”) on a 4-point Likert-like scale ranging from “*Never*“ to “*Often*”.

## Tables

|  | T1 (17y, 1994) | T2 (22y, 1999) | T3 (28y, 2005) | T4 (43y, 2020) |
| --- | --- | --- | --- | --- |
| Current smoking, n (%) | 802 (26.11) | 1091 (35.51) | 834 (27.15) | 253 (8.24) |
| Alcohol use, mean (SD) | 13.18 (32.06) | 28.25 (49.62) | 24.88 (46.29) | 14.71 (28.78) |
| Body mass index, mean (SD) | 20.94 (2.74) | 22.98 (3.42) | 24.37 (4.08) | 26.18 (4.70) |
| Loneliness, mean (SD) | 1.78 (0.55) | 1.72 (0.49) | 1.70 (0.49) | 1.80 (0.55) |
| Physical exercise, mean (SD) | 176.93 (173.12) | 176.73 (259.84) | 146.46 (195.62) | 196.79 (177.56) |

Table S1. Wave-specific descriptive statistics. *Note:* Statistics are based on the incomplete dataset before multiple imputations.

|  | T1 (17y, 1994) | T2 (22y, 1999) | T3 (28y, 2005) | T4 (43y, 2020) |
| --- | --- | --- | --- | --- |
| Alcohol use (drinks per 4 weeks) | 0 to 300 | 0 to 300 | 0 to 300 | 0 to 290 |
| Body mass index (kg/m^2^) | 13 to 50 | 9 to 46 | 16 to 58 | 13 to 56 |
| Loneliness (composite mean score) | 1 to 4 | 1 to 4 | 1 to 4 | 1 to 4 |
| Physical exercise minutes per week | 0 to 1260 | 0 to 2400 | 0 to 2400 | 0 to 2250 |

Table S2. Wave-specific scale ranges (min to max) for exposure and outcome measures. *Note:* Statistics are based on the incomplete dataset before multiple imputations.

|  | T0 (15y, 1992) | T1 (17y, 1994) | T2 (22y, 1999) | T3 (28y, 2005) | T4 (43y, 2020) |
| --- | --- | --- | --- | --- | --- |
| Age | 0 |  |  |  |  |
| Gender | 0 |  |  |  |  |
| Ethnicity | 2 |  |  |  |  |
| Chronic condition | 0 |  |  |  |  |
| Parental education | 4 |  |  |  |  |
| Behavioral monitoring | 2 |  |  |  |  |
| Psychological overcontrol | 2 |  |  |  |  |
| Cold parenting | 2 |  |  |  |  |
| Parental alcohol use | 3 |  |  |  |  |
| Parental smoking | 12 |  |  |  |  |
| Social support | 7 | 1 | 10 |  |  |
| Depressive symptoms | 3 | 5 | 12 |  |  |
| Education |  |  | 0 |  |  |
| Employment | 2 | 1 | 12 |  |  |
| Living situation | 2 | 0 | 11 |  |  |
| Relationship | 0 | 0 | 10 |  |  |
| Friends | 2 | 4 | 11 |  |  |
| Smoking | 4 | 5 | 14 | 11 | 33 |
| Alcohol use | 6 | 6 | 14 | 16 | 35 |
| Physical exercise | 1 | 8 | 14 | 13 | 34 |
| Body mass index | 4 | 2 | 13 | 12 | 33 |
| Loneliness | 4 | 4 | 11 | 11 | 32 |

Table S3. Proportions (%) of missing data by variable and data collection wave.

|  |  | Late adolescence (17y, 1994) | Emerg. adulthood (22y, 1999) | Young adulthood (28y, 2005) |
| --- | --- | --- | --- | --- |
| **Alcohol use** (drinks per 4 weeks) | **Abstinence m** | 0 | 0 | 0 |
|  | **Moderate** (mean) | 12.5 | 26.2 | 23.4 |
|  | **Heavy** (1SD above mean) | 38.9 | 64.2 | 58.3 |
| **Body mass index** (kg/m^2^) | **Underweight** (1SD below mean) | 18.2 | 19.6 | 20.3 |
|  | **Normative** (mean) | 20.9 | 23.0 | 24.4 |
|  | **Overweight** (1SD above mean) | 23.7 | 26.4 | 28.5 |
| **Physical exercise** (minutes per week) | **Inactivity** | 0 | 0 | 0 |
|  | **Moderate** (mean) | 176.9 | 176.7 | 146.5 |

Table S4. Life stage-specific exposure contrasts for continuous health behaviors.

## Figures

Figure S1. Flowchart of the study participation from T0 (1992) to T4 (2020).

Figure S2. Directed acyclic graph (DAG) representing the assumed causal structure of exposures (X_0_-X_4_), outcomes (Y_0_-Y_4_), time-varying confounders (L_0_-L_4_), and baseline confounders (C_0_) across five data collection waves from adolescence (T0) to midlife (T4). Red lines indicate causal paths of interest. Separately for each health behavior, we fitted two series of structural mean models – one with loneliness as exposure and health behavior as outcome, and the second with health behavior as exposure and loneliness as outcome. *Note*: C_0_ is assumed to influence all other variables.

Figure S3. Specification of the exposure model (panel A) and outcome model (panel B). Adapted from Mulder et al. (2024)^10^.

Figure S4. Estimates for gender-specific effects of loneliness on health behaviors. *Note:* Labels of life stages below the panels refer to the exposure period.

Figure S5. Estimates for gender-specific effects of health behaviors on loneliness. *Note:* Labels of life stages below the panels refer to the exposure period.

Figure S6. Estimates for effects of loneliness on health behaviors, unadjusted for time-varying indicators of social support, relationship status, living situation, and number of friends. *Note:* Labels of life stages below the panels refer to the exposure period.

Figure S7. Estimates for effects of loneliness on health behaviors, unadjusted for time-varying indicators of depressive symptoms, social support, relationship status, living situation, and number of friends. *Note:* Labels of life stages below the panels refer to the exposure period.

Figure S8. Estimates for effects of binge drinking (heavy episodic drinking) on loneliness. *Note:* Labels of life stages below the panels refer to the exposure period.

Figure S9. Estimates for effects of health behaviors on loneliness with life-stage specific contrasts. *Note:* Labels of life stages below the panels refer to the exposure period.

Figure S10. Estimates for effects of loneliness measured directly on health behaviors. *Note:* Labels of life stages below the panels refer to the exposure period.

Figure S11. Estimates for effects of health behaviors on loneliness measured directly. *Note:* Labels of life stages below the panels refer to the exposure period.

Figure S12. Estimates for bi-directional effects of loneliness and depressive symptoms. *Note:* Labels of life stages below the panels refer to the exposure period.

Figure S13. Bivariate associations between loneliness and subsequent health behaviors. *Note:* Labels of life stages below the panels refer to the exposure period.

Figure S14. Directed acyclic graph of unmeasured confounding, from VanderWeele (2019). *Note*: As demonstrated by VanderWeele (2019), adjusting for measured confounders C that are downstream of unmeasured causes can still reduce confounding between the exposure A and outcome Y, even when the unmeasured confounder U is not directly observed.

**References**

1	Pitt TM, Hetherington E, Adhikari K, *et al.* Developing non-response weights to account for attrition-related bias in a longitudinal pregnancy cohort. *BMC Med Res Methodol* 2023; **23**: 295.

2	Schmidt SCE, Woll A. Longitudinal drop-out and weighting against its bias. *BMC Med Res Methodol* 2017; **17**: 164.

3	Walter S, Tiemeier H. Variable selection: current practice in epidemiological studies. *Eur J Epidemiol* 2009; **24**: 733–6.

4	Lee BK, Lessler J, Stuart EA. Weight Trimming and Propensity Score Weighting. *PLOS ONE* 2011; **6**: e18174.

5	Potočár L, Kozák M, Bøe T, von Soest T. The social gradients in mental health and psychosocial well-being from adolescence to midlife and the mediating role of parenting practices: a national cohort study. *Social Science & Medicine* 2025; **383**: 118405.

6	Parker G, Tupling H, Brown LB. A parental bonding instrument. *Br J Med Psychol* 1979; **52**: 1–10.

7	Pedersen W. Parental relations, mental health, and delinquency in adolescents. *Adolescence* 1994; **29**: 975–90.

8	Sarason IG, Sarason BR, Shearin EN, Pierce GR. A Brief Measure of Social Support: Practical and Theoretical Implications. *J Soc Pers Relat* 1987; **4**: 497–510.

9	Kandel DB, Davies M. Epidemiology of depressive mood in adolescents: an empirical study. *Arch Gen Psychiatry* 1982; **39**: 1205–12.

10	Mulder JD, Usami S, Hamaker EL. Joint Effects in Cross-Lagged Panel Research Using Structural Nested Mean Models. *Structural Equation Modeling: A Multidisciplinary Journal* 2024; **0**: 1–17.

11	Visontay R, Mewton L, Slade T, Aris IM, Sunderland M. Moderate Alcohol Consumption and Depression: A Marginal Structural Model Approach Promoting Causal Inference. *AJP* 2023; **180**: 209–17.

12	Desquilbet L, Mariotti F. Dose-response analyses using restricted cubic spline functions in public health research. *Stat Med* 2010; **29**: 1037–57.

13	Ho FK, Cole TJ. Non-linear predictor outcome associations. *bmjmed* 2023; **2**. DOI:10.1136/bmjmed-2022-000396.

14	Bailey DH, Jung AJ, Beltz AM, *et al.* Causal inference on human behaviour. *Nat Hum Behav* 2024; **8**: 1448–59.

15	Mulder JD, Usami S, Hamaker EL. Joint Effects in Cross-Lagged Panel Research Using Structural Nested Mean Models. *Structural Equation Modeling: A Multidisciplinary Journal* 2024; **0**: 1–17.

16	Picciotto S, Neophytou AM. G-Estimation of Structural Nested Models: Recent Applications in Two Subfields of Epidemiology. *Curr Epidemiol Rep* 2016; **3**: 242–51.

17	Vansteelandt S, Goetghebeur E. Causal Inference with Generalized Structural Mean Models. *Journal of the Royal Statistical Society Series B: Statistical Methodology* 2003; **65**: 817–35.

18	Rehkopf DH, Glymour MM, Osypuk TL. The Consistency Assumption for Causal Inference in Social Epidemiology: When a Rose Is Not a Rose. *Curr Epidemiol Rep* 2016; **3**: 63–71.

19	VanderWeele TJ. Principles of confounder selection. *Eur J Epidemiol* 2019; **34**: 211–9.

20	Vansteelandt S, Joffe M. Structural Nested Models and G-estimation: The Partially Realized Promise. *Statistical Science* 2014; **29**: 707–31.

21	Yang S. Semiparametric Estimation of Structural Nested Mean Models with Irregularly Spaced Longitudinal Observations. *Biometrics* 2022; **78**: 937–49.

22	National Institute on Alcohol Abuse and Alcoholism. Drinking Levels and Patterns Defined | National Institute on Alcohol Abuse and Alcoholism (NIAAA). 2024. https://www.niaaa.nih.gov/alcohol-health/overview-alcohol-consumption/moderate-binge-drinking (accessed Dec 2, 2024).

23	Nicolaisen M, Thorsen K. Who are lonely? Loneliness in different age groups (18-81 years old), using two measures of loneliness. *Int J Aging Hum Dev* 2014; **78**: 229–57.
